# Design and Vibration Sensitivity Analysis of a MEMS Tuning Fork Gyroscope with an Anchored Diamond Coupling Mechanism

**DOI:** 10.3390/s16040468

**Published:** 2016-04-02

**Authors:** Yanwei Guan, Shiqiao Gao, Haipeng Liu, Lei Jin, Shaohua Niu

**Affiliations:** 1 State Key Laboratory of Explosion Science and Technology, Beijing Institute of Technology, Beijing 100081, China; guanyanwei2006@163.com (Y.G.); gaoshq@bit.edu.cn (S.G.); shh@bit.edu.cn (S.N.); 2 School of Mechatronical Engineering, Beijing Institute of Technology, Beijing 100081, China; jinlei@bit.edu.cn

**Keywords:** vibration sensitivity, tuning fork gyroscopes, anchored diamond coupling mechanism, stiffness difference ratio, coordinate transformation method

## Abstract

In this paper, a new micromachined tuning fork gyroscope (TFG) with an anchored diamond coupling mechanism is proposed while the mode ordering and the vibration sensitivity are also investigated. The sense-mode of the proposed TFG was optimized through use of an anchored diamond coupling spring, which enables the in-phase mode frequency to be 108.3% higher than the anti-phase one. The frequencies of the in- and anti-phase modes in the sense direction are 9799.6 Hz and 4705.3 Hz, respectively. The analytical solutions illustrate that the stiffness difference ratio of the in- and anti-phase modes is inversely proportional to the output induced by the vibration from the sense direction. Additionally, FEM simulations demonstrate that the stiffness difference ratio of the anchored diamond coupling TFG is 16.08 times larger than the direct coupling one while the vibration output is reduced by 94.1%. Consequently, the proposed new anchored diamond coupling TFG can structurally increase the stiffness difference ratio to improve the mode ordering and considerably reduce the vibration sensitivity without sacrificing the scale factor.

## 1. Introduction

The operational principle of MEMS vibrating gyroscopes actually relies on the Coriolis effect with an energy transfer between two vibration modes [1]. Typically, a micromachined gyroscope is a resonator with a drive-mode and a sense-mode. With the improvement in the performance specifications of MEMS gyroscopes such as the resolution, sensitivity, and bandwidth [2,3,4,5,6], external vibrations can significantly influence the gyroscope’s sensitivity due to the high quality factor which ranges from a hundred or so at atmospheric pressure to hundreds of thousands under vacuum.

A dual-mass tuning fork gyro is a very common type of vibrating gyroscope used to cancel linear vibrations by using two identical tines, which operate in anti-phase mode [7,8,9,10]. The linear vibration is a common-mode vibration caused by random environment vibrations. Due to the inevitable structural imbalance, the linear vibration will cause output errors. To decrease the vibration output caused by fabrication defects, a large frequency separation between the in- and anti-phase modes is needed [11,12,13]. However, these methods reduce the scale factor of TFGs. To resist the linear vibration without sacrificing the sensitivity and eliminate the lower frequency mode, an improved mode ordering by using different coupling mechanisms between two tines is necessary [14,15,16]. Therefore, shifting the in-phase mode frequency above the anti-phase and increasing the frequency separation are important.

In our previous work [16], we designed a dual-mass TFG with an anchored ring coupling mechanism and only numerically studied the linear vibration sensitivity of proposed TFGs due to the stiffness imbalance induced by the practical process imperfections. It was observed that linear vibration would induce output errors in the in- and anti-phase mode frequencies.

This paper proposes a new micromachined TFG with an anchored diamond coupling beam to analyze the mode ordering and the vibration sensitivity. For comparison with this novel structure, a traditional one with a direct diamond coupling beam was designed at the same time. Section 2 describes the two kinds of structures in more detail. An analytical analysis on the response of the non-ideal TFG with anchored coupling is given in Section 3. FEM simulations on the stiffness of different coupling styles and the comparisons with simulations and analytical solutions are presented in Section 4. In Section 5, possible methods to decrease the vibration sensitivity and their principles are discussed. Section 6 gives our conclusions.

## 2. Architecture Design

**Figure 1:**
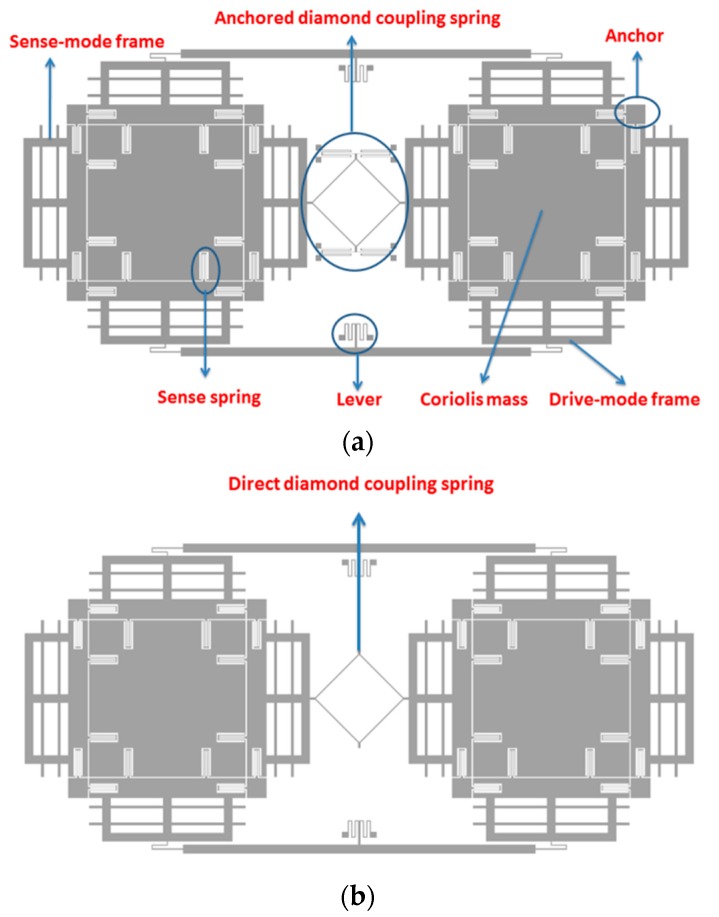
Schematic of the designed TFGs (**a**) Type A (**b**) Type B.

Two kinds of TFGs are designed in this paper according to the previous work [16]. The type A architecture is a dual-mass structure where the left mass and right mass are symmetrical, as depicted in Figure 1a, which shows two identical masses, a lever beam and an anchored diamond coupling spring. The architecture of type B is the same with as that of type A, except for the fact that the coupling type, which is direct coupling via a diamond spring, is different, as depicted in Figure 1b.

**Figure 2:**
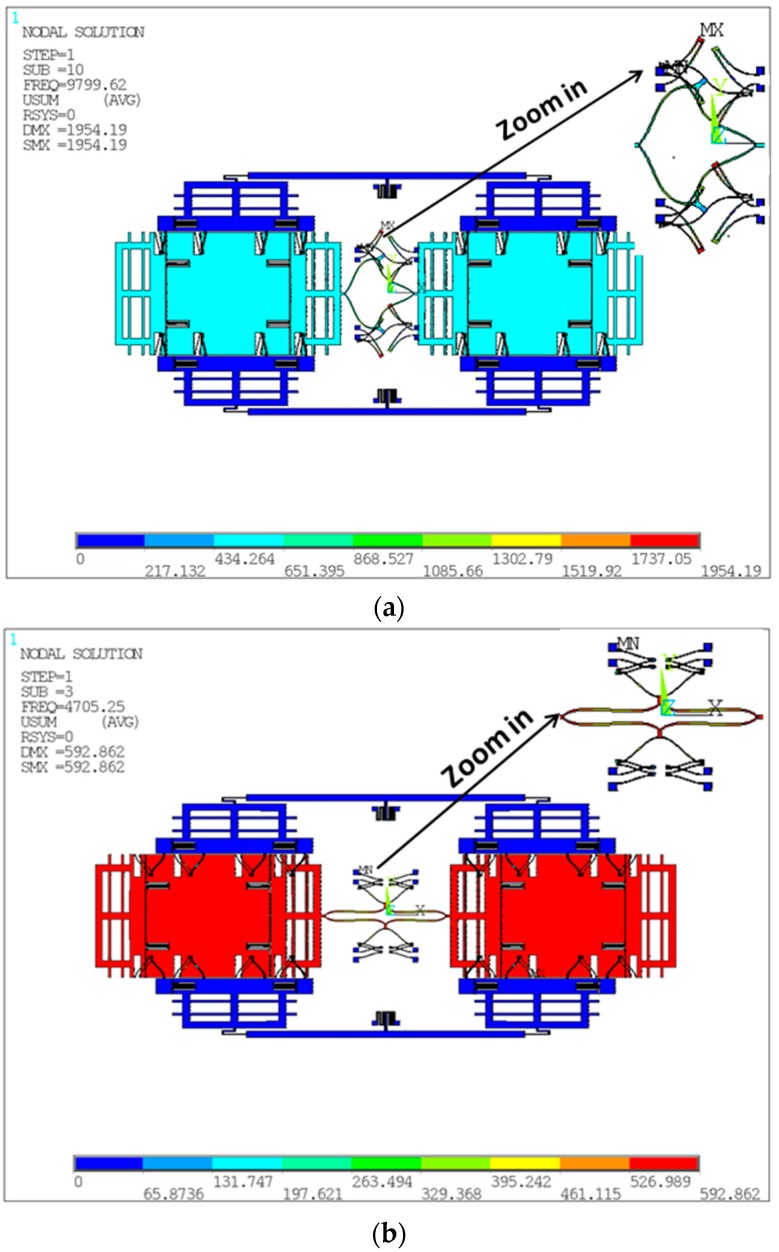
Modal analysis of the in-phase mode (**a**) and anti-phase mode (**b**) of Type A.

**Figure 3:**
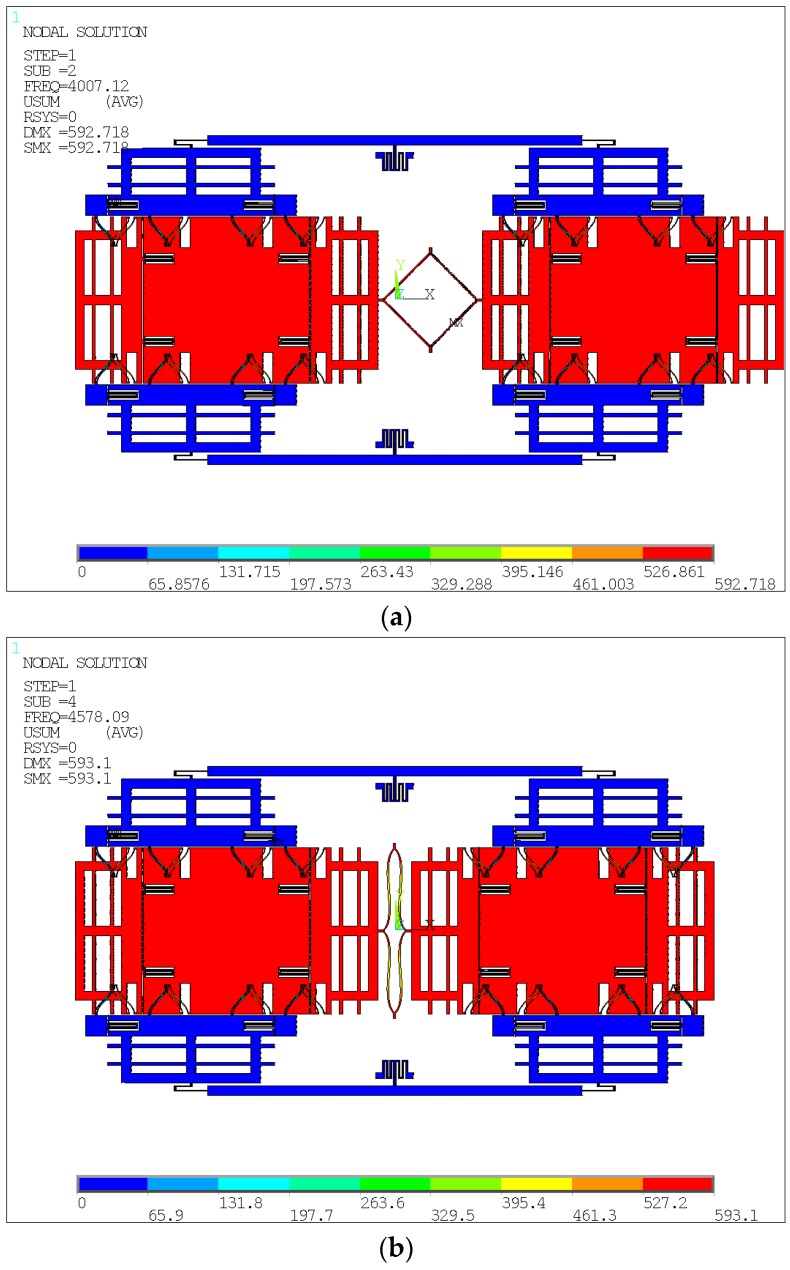
Modal analysis of the in-phase mode (**a**) and anti-phase mode (**b**) of Type B.

Every tine includes a Coriolis mass and two frames, supported by symmetrical springs. The springs, except the supporting lever springs and the anchored coupling ones, are the same to increase the robustness of the mode-match between drive and sense modes and to resist any temperature shifts of the resonance frequencies. The electrodes are variable-area capacitances to guarantee the linearity of the capacitance change with the displacement in the motion direction parallel to the plates. The mode analysis of two types in the in- and anti-phase modes in the sense direction is carried out and shown in Figure 2a,b and Figure 3a,b, respectively. It is demonstrated that the Type A architecture can optimize the modal order. Specifically, the in-phase mode frequency is improved by nearly 110% compared with the anti-phase. Therefore, the Type A structure offers a new architecture to truly reject the external shock and vibration by making the in-phase modal frequency larger than the anti-phase.

## 3. Theoretical Analysis

**Figure 4:**
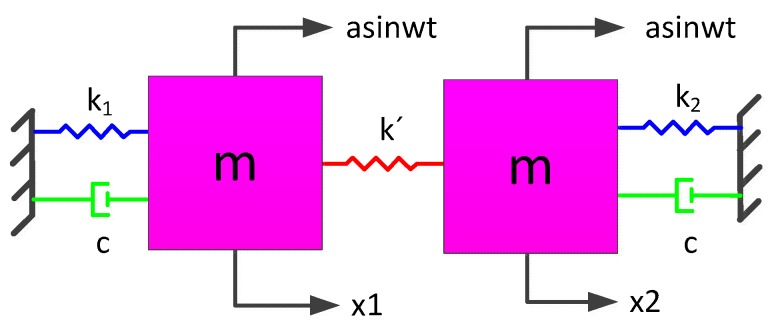
The model of the non-ideal TFG.

First, the non-ideal TFG model is shown in Figure 4. The dynamics are governed by:

Left mass:

(1) $$m{\ddot{x}}_{1}+c{\dot{x}}_{1}+k_{1}x_{1}+k^{\prime }(x_{1}-x_{2})=ma\sin wt$$

Right mass:

(2) $$m{\ddot{x}}_{2}+c{\dot{x}}_{2}+k_{2}x_{2}+k^{\prime }(x_{2}-x_{1})=ma\sin wt$$

Subtracting Equation (2) from Equation (1), one obtains:

(3) $$m{\ddot{x}}_{1}+c{\dot{x}}_{1}+k_{1}x_{1}+k^{\prime }(x_{1}-x_{2})-m{\ddot{x}}_{2}-c{\dot{x}}_{2}-k_{2}x_{2}-k^{\prime }(x_{2}-x_{1})=0$$

Adding Equations (1) and (2), one obtains:

(4) $$m{\ddot{x}}_{1}+c{\dot{x}}_{1}+k_{1}x_{1}+m{\ddot{x}}_{2}+c{\dot{x}}_{2}+k_{2}x_{2}=2ma\sin wt$$

Here, *m* and *c* are the mass and damping of each tine respectively, *k*_1_ and *k*_2_ denote the stiffness and *k*’ is the coupling stiffness in the anti-phase mode, *x*_1_ and *x*_2_ are the displacement, *a*sin*wt* is the external acceleration acting on the whole TFG, in which *a* denotes the amplitude and *w* is the angular frequency.

Since the vibration output of in- and anti-phase modes needs to be analyzed, a coordinate transformation is made as follows:

(5) $$x_{an}=x_{1}-x_{2},\;x_{in}=x_{1}+x_{2}$$

Substituting Equation (5) into Equations (3) and (4), one obtains:

(6) $$\begin{array}{c} {\ddot{x}}_{an}+\frac{w_{an}}{Q_{an}}{\dot{x}}_{an}+w_{an}^{2}x_{an}=-\frac{\Delta k}{2m}x_{in}\\ {\ddot{x}}_{in}+\frac{w_{in}}{Q_{in}}{\dot{x}}_{in}+w_{in}^{2}x_{in}=-\frac{\Delta k}{2m}x_{an}+2a\sin wt \end{array}$$

where $w_{an}=\sqrt{\frac{k_{1}+k_{2}+4k^{\prime }}{2m}}$, $Q_{an}=\frac{mw_{an}}{c}$, $w_{in}=\sqrt{\frac{k_{1}+k_{2}}{2m}}$, $Q_{in}=\frac{mw_{in}}{c}$, $k_{1}-k_{2}=\Delta k$. In which, $w_{an}$ and $w_{in}$ are the defined resonant frequencies in the anti- and in-phase modes, $Q_{an}$ and $Q_{in}$ are quality factors of the ideal anti- and in-phase motions and Δk is the stiffness imbalance.

The Equation (6) can be expressed as a matrix representation:

(7) $$M\overset{¨}{x}+C\dot{x}+Kx=F\sin wt$$

where: $M=\left[\begin{array}{cc} 1 & 0\\ 0 & 1 \end{array}\right]$, $C=\left[\begin{array}{cc} \frac{w_{an}}{Q_{an}} & 0\\ 0 & \frac{w_{in}}{Q_{in}} \end{array}\right]$, $K=\left[\begin{array}{cc} w_{an}^{2} & \frac{\Delta k}{2m}\\ \frac{\Delta k}{2m} & w_{in}^{2} \end{array}\right]$, $F=\left[\begin{array}{c} 0\\ 2a \end{array}\right]$, $x=\left[\begin{array}{c} x_{an}\\ x_{in} \end{array}\right]$

The natural frequency can be obtained by using the characteristic equation:

(8) $$\begin{array}{c} w_{1}^{2}=\frac{\left(w_{in}^{2}+w_{an}^{2}\right)-\sqrt{{\left(w_{in}^{2}-w_{an}^{2}\right)}^{2}+{\left(\frac{\Delta k}{m}\right)}^{2}}}{2}\\ w_{2}^{2}=\frac{\left(w_{in}^{2}+w_{an}^{2}\right)+\sqrt{{\left(w_{in}^{2}-w_{an}^{2}\right)}^{2}+{\left(\frac{\Delta k}{m}\right)}^{2}}}{2} \end{array}$$

where $w_{1}$ is the first-order resonant frequency and $w_{2}$ is the second-order resonant frequency.

The modal superposition technique is used to acquire the steady-state response through solving Equation (7):

(9) $$x(t)=\frac{2\beta_{1}a}{w_{1}^{2}}\cdot \frac{1}{1+{\left(\frac{\Delta k/m}{\sqrt{{\left(w_{in}^{2}-w_{an}^{2}\right)}^{2}+{\left(\frac{\Delta k}{m}\right)}^{2}}+\left(w_{in}^{2}-w_{an}^{2}\right)}\right)}^{2}}\cdot \left[\begin{array}{c} \frac{-\Delta k/m}{\sqrt{{\left(w_{in}^{2}-w_{an}^{2}\right)}^{2}+{\left(\frac{\Delta k}{m}\right)}^{2}}+\left(w_{in}^{2}-w_{an}^{2}\right)}\\ {\left(\frac{\Delta k/m}{\sqrt{{\left(w_{in}^{2}-w_{an}^{2}\right)}^{2}+{\left(\frac{\Delta k}{m}\right)}^{2}}+\left(w_{in}^{2}-w_{an}^{2}\right)}\right)}^{2} \end{array}\right]+\;\frac{2\beta_{2}a}{w_{2}^{2}}\cdot \frac{1}{1+{\left(\frac{\Delta k/m}{\sqrt{{\left(w_{in}^{2}-w_{an}^{2}\right)}^{2}+{\left(\frac{\Delta k}{m}\right)}^{2}}-\left(w_{in}^{2}-w_{an}^{2}\right)}\right)}^{2}}\cdot \left[\begin{array}{c} \frac{\Delta k/m}{\sqrt{{\left(w_{in}^{2}-w_{an}^{2}\right)}^{2}+{\left(\frac{\Delta k}{m}\right)}^{2}}-\left(w_{in}^{2}-w_{an}^{2}\right)}\\ {\left(\frac{\Delta k/m}{\sqrt{{\left(w_{in}^{2}-w_{an}^{2}\right)}^{2}+{\left(\frac{\Delta k}{m}\right)}^{2}}-\left(w_{in}^{2}-w_{an}^{2}\right)}\right)}^{2} \end{array}\right]$$

where, the magnification factor of amplitudes $\beta_{i}=\frac{1}{\sqrt{{\left(1-\lambda_{i}^{2}\right)}^{2}+{\left(2\xi_{i}\lambda_{i}\right)}^{2}}}$, the phase angle $\psi_{i}=\arctan\frac{2\xi_{i}\lambda_{i}}{1-\lambda_{i}^{2}}$, the frequency ratio $\lambda_{i}=\frac{w}{w_{i}}$, and the damping ratio $\xi_{i}=\frac{c}{2w_{i}m}$.

When $w=w_{1}$, we can obtain that:

(10) $$x_{an}(t)=\frac{2Q_{1}a}{w_{1}^{2}}\cdot \frac{\frac{-\Delta k/m}{\sqrt{{\left(w_{in}^{2}-w_{an}^{2}\right)}^{2}+{\left(\frac{\Delta k}{m}\right)}^{2}}+\left(w_{in}^{2}-w_{an}^{2}\right)}}{1+{\left(\frac{\Delta k/m}{\sqrt{{\left(w_{in}^{2}-w_{an}^{2}\right)}^{2}+{\left(\frac{\Delta k}{m}\right)}^{2}}+\left(w_{in}^{2}-w_{an}^{2}\right)}\right)}^{2}}=\frac{2Q_{1}a}{w_{1}^{2}}\cdot \frac{\frac{-\Delta k}{\sqrt{{\left(\frac{k_{in}-k_{an}}{2}\right)}^{2}+{\left(\Delta k\right)}^{2}}+\left(\frac{k_{in}-k_{an}}{2}\right)}}{1+{\left(\frac{\Delta k}{\sqrt{{\left(\frac{k_{in}-k_{an}}{2}\right)}^{2}+{\left(\Delta k\right)}^{2}}+\left(\frac{k_{in}-k_{an}}{2}\right)}\right)}^{2}}$$

where the anti-phase stiffness is $k_{an}=k_{1}+k_{2}+4k^{\prime }$, and the in-phase stiffness is $k_{in}=k_{1}+k_{2}$.

Considering the actual fabrication defects, $k_{an}-k_{in}>>\Delta k$ and using the Taylor series expansion:

$$\frac{\Delta k}{\sqrt{{\left(\frac{k_{in}-k_{an}}{2}\right)}^{2}+{\left(\Delta k\right)}^{2}}+\left(\frac{k_{in}-k_{an}}{2}\right)}=\frac{k_{an}-k_{in}}{\Delta k}$$

so Equation (10) can be rewritten as:

(11) $$x_{{}_{an}}^{\left(1\right)}(t)=\frac{2Q_{1}a}{w_{1}^{2}}\cdot \frac{\Delta k}{k_{in}-k_{an}}\cos w_{1}t=\frac{2Q_{1}a}{w_{1}^{2}}\cdot \frac{\Delta k}{k}\cdot \frac{k}{k_{an}-k_{in}}\cos w_{1}t$$

where $Q_{1}$ is the first-order mode Q-factor.

When $w=w_{2}$, we can obtain that:

(12) $$x_{an}(t)=\frac{2Q_{2}a}{w_{2}^{2}}\cdot \frac{\frac{\Delta k/m}{\sqrt{{\left(w_{in}^{2}-w_{an}^{2}\right)}^{2}+{\left(\frac{\Delta k}{m}\right)}^{2}}-\left(w_{in}^{2}-w_{an}^{2}\right)}}{1+{\left(\frac{\Delta k/m}{\sqrt{{\left(w_{in}^{2}-w_{an}^{2}\right)}^{2}+{\left(\frac{\Delta k}{m}\right)}^{2}}-\left(w_{in}^{2}-w_{an}^{2}\right)}\right)}^{2}}=\frac{2Q_{2}a}{w_{2}^{2}}\cdot \frac{\frac{\Delta k}{\sqrt{{\left(\frac{k_{in}-k_{an}}{2}\right)}^{2}+{\left(\Delta k\right)}^{2}}-\left(\frac{k_{in}-k_{an}}{2}\right)}}{1+{\left(\frac{\Delta k}{\sqrt{{\left(\frac{k_{in}-k_{an}}{2}\right)}^{2}+{\left(\Delta k\right)}^{2}}-\left(\frac{k_{in}-k_{an}}{2}\right)}\right)}^{2}}$$

where $Q_{2}$ is the second-order mode Q-factor.

Considering that $k_{an}-k_{in}>>\Delta k$ and by the Taylor series expansion:

$$\frac{\Delta k}{\sqrt{{\left(\frac{k_{in}-k_{an}}{2}\right)}^{2}+{\left(\Delta k\right)}^{2}}-\left(\frac{k_{in}-k_{an}}{2}\right)}=\frac{\Delta k}{k_{an}-k_{in}}$$

so, Equation (12) can be rewritten as:

(13) $$x_{{}_{an}}^{\left(2\right)}(t)=\frac{2Q_{2}a}{w_{2}^{2}}\cdot \frac{\Delta k}{k_{in}-k_{an}}\cos w_{2}t=\frac{2Q_{2}a}{w_{2}^{2}}\cdot \frac{\Delta k}{k}\cdot \frac{k}{k_{an}-k_{in}}\cos w_{2}t$$

The traditional direct coupling beam has no deformation in the in-phase mode, so $k_{in}$ is $k_{1}+k_{2}$ and $w_{in}$ is $\sqrt{\frac{k_{1}+k_{2}}{2m}}$. However, the deformation styles of the anchored coupling beam are different in the in- and anti-phase modes. In the anti-phase, the beam is bent under a lateral load while the beam is tensile (compressed) under an axial load in the in-phase. So, the stiffness of the anchored coupling beam in the in-phase mode is added to the in-phase stiffness, and $k_{in}$ is $k_{1}+k_{2}+2k^{\prime \prime }$, and $k^{\prime \prime }$ is the coupling stiffness in the in-phase mode and $w_{in}$ is $\sqrt{\frac{k_{1}+k_{2}+2k^{\prime \prime }}{2m}}$.

Since the in-phase stiffness is different between the direct coupling and anchored coupling TFGs, the vibration output in the anchored coupling structure should be recalculated. Considering the actual fabrication defects, $k_{in}-k_{an}>>\Delta k$ and using the Taylor series expansion:

$$\frac{\Delta k}{\sqrt{{\left(\frac{k_{in}-k_{an}}{2}\right)}^{2}+{\left(\Delta k\right)}^{2}}+\left(\frac{k_{in}-k_{an}}{2}\right)}=\frac{\Delta k}{k_{in}-k_{an}}$$

$$\frac{\Delta k}{\sqrt{{\left(\frac{k_{in}-k_{an}}{2}\right)}^{2}+{\left(\Delta k\right)}^{2}}-\left(\frac{k_{in}-k_{an}}{2}\right)}=\frac{k_{in}-k_{an}}{\Delta k}$$

so, Equation (10) can be rewritten as:

(14) $$x_{{}_{an}}^{\left(1\right)}(t)=\frac{2Q_{1}a}{w_{1}^{2}}\cdot \frac{\Delta k}{k_{in}-k_{an}}\cos w_{1}t=\frac{2Q_{1}a}{w_{1}^{2}}\cdot \frac{\Delta k}{k}\cdot \frac{k}{k_{in}-k_{an}}\cos w_{1}t$$

Equation (12) can be rewritten as:

(15) $$x_{{}_{an}}^{\left(2\right)}(t)=\frac{2Q_{2}a}{w_{2}^{2}}\cdot \frac{\Delta k}{k_{in}-k_{an}}\cos w_{2}t=\frac{2Q_{2}a}{w_{2}^{2}}\cdot \frac{\Delta k}{k}\cdot \frac{k}{k_{in}-k_{an}}\cos w_{2}t$$

Here, the dimensionless parameters ε and η are defined. ε denotes the stiffness imbalance, and η denotes the ratio of the stiffness difference between in- and anti-phase modes to the stiffness k in the sense direction, which is defined as the stiffness difference ratio (SDR). The two parameters are given by:

$$\varepsilon =\frac{\Delta k}{k},\;\eta =\frac{\left|k_{an}-k_{in}\right|}{k}$$

Then, Equations (11) and (14) can be expressed as:

(16) $$x_{{}_{an}}^{\left(1\right)}(t)=\frac{2Q_{1}a}{w_{1}^{2}}\cdot \frac{\varepsilon }{\eta }\cdot \cos w_{1}t$$

Equations (13) and (15) can be expressed as:

(17) $$x_{{}_{an}}^{\left(2\right)}(t)=\frac{2Q_{2}a}{w_{2}^{2}}\cdot \frac{\varepsilon }{\eta }\cdot \cos w_{2}t$$

From Equations (16) and (17), it is figured out that the anti-phase vibration output is proportional to ε and inversely proportional to the stiffness difference ratio η.

## 4. FEM Simulations

### 4.1. SDR Analysis

First, the SDR of Type A and Type B TFGs, which are represented by $\eta_{a},\eta_{b}$, respectively, are analyzed. According to the definition of the stiffness difference ratio η:

(18) $$\eta_{a}=\frac{\left|k_{in}-k_{an}\right|}{k}=\frac{2k^{\prime \prime }-4k^{\prime }}{k},\;\eta_{b}=\frac{\left|k_{in}-k_{an}\right|}{k}=\frac{4k^{\prime }}{k}$$

**Figure 5:**
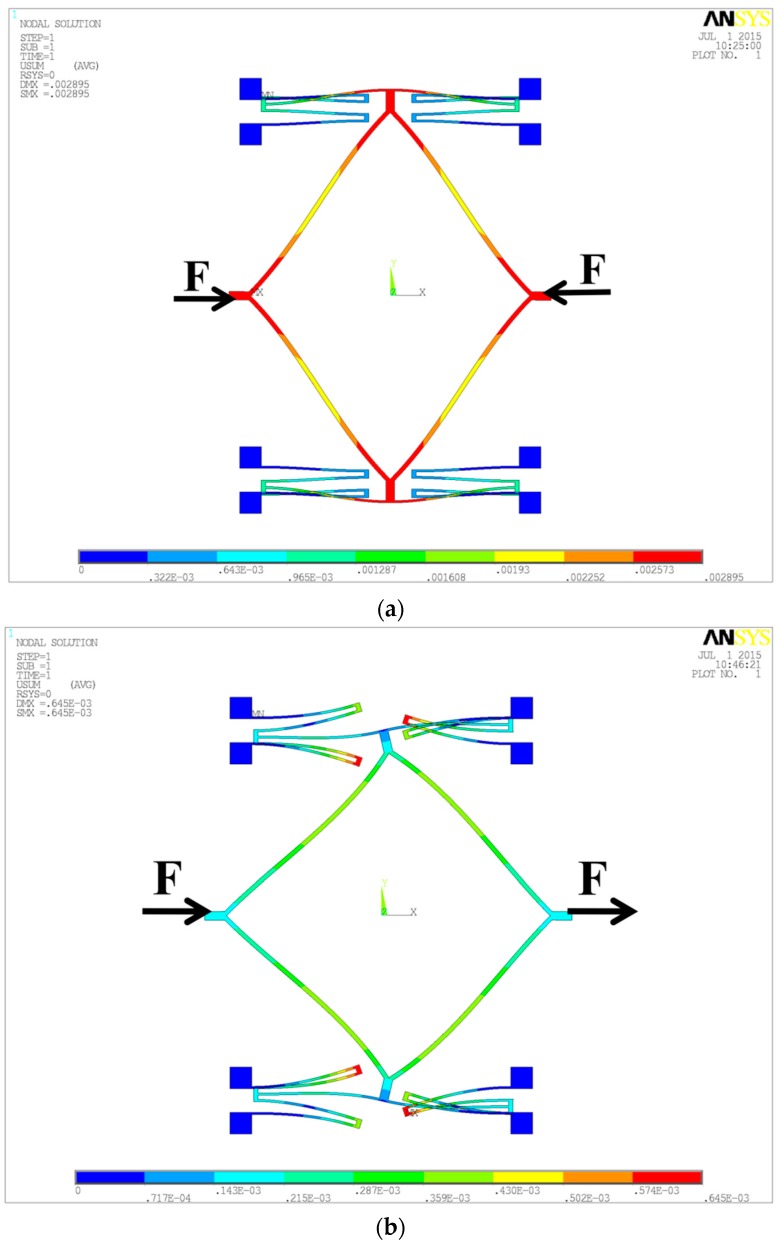
The stiffness of the anchored diamond coupling beam in the anti-phase mode (**a**) and in-phase mode (**b**).

From Equation (18), the stiffness difference is dependent on the in- and anti-phase coupling stiffness, so simulations are carried out on the stiffness of the coupling spring by applying a 1 μN force in the sense direction, shown in Figure 5. The length of the linear beam is 500 μm and the width is 10 μm while the length of the diamond beam is 1000 μm and the width is 20 μm. The anti- and in-phase coupling stiffness of the anchored diamond coupling structure are shown in Figure 5a and Figure 5b, respectively. By the formula *k* = *F*/*x*, the stiffness *k* can be obtained:

(19) $$2k_{a}^{\prime }=\frac{1\mu N}{0.002895\mu m}=345.42\text{ N}/m,\;k_{a}^{\prime \prime }=\frac{1\mu N}{0.21\times 10^{-3}\mu m}=4761.90\text{ N}/m$$

Substituting Equation (19) into Equation (18), we can obtain:

(20) $$\eta_{a}=\frac{2k_{a}^{\prime \prime }-4k_{a}^{\prime }}{k}=11.80$$

**Figure 6:**
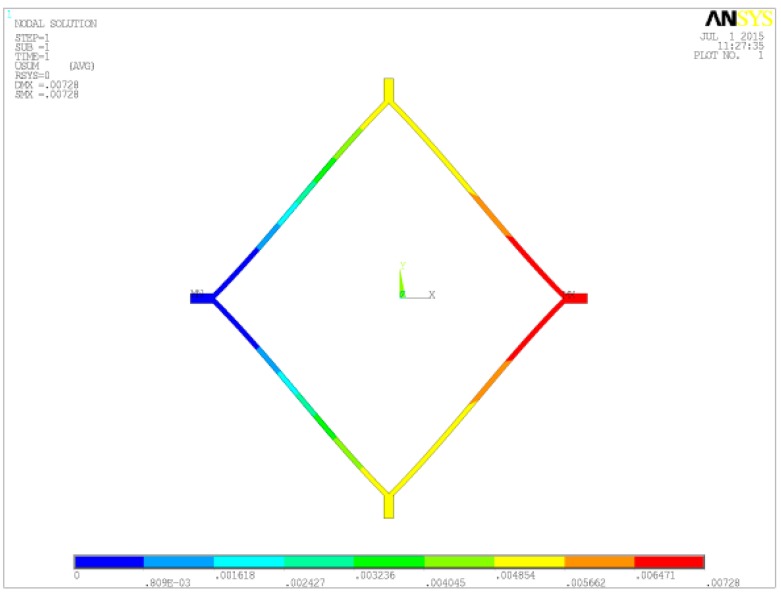
The stiffness of the direct diamond coupling beam in the anti-phase mode.

The anti-phase coupling stiffness of the direct diamond coupling structure is shown in Figure 6. The stiffness k can be obtained from:

(21) $$k_{b}^{\prime }=\frac{1\mu N}{0.00728\mu m}=137.36\;N/m$$

Substituting Equation (21) into Equation (18), we can obtain:

(22) $$\eta_{b}=\frac{4k_{b}^{\prime }}{k}=0.73$$

Therefore, the ratio of SDR of the anchored and direct diamond coupling structures can be computed from Equations (20) and (22):

(23) $$\frac{\eta_{a}}{\eta_{b}}=\frac{2k_{a}^{\prime \prime }-4k_{a}^{\prime }}{4k_{b}^{\prime }}=16.08$$

Similarly, the SDR of the anchored ring coupling structure in our previous study [16] is studied and it is 2.16. Therefore, the ratio of SDR of the anchored diamond and ring coupling structures is 5.46. From the above numerical analysis, it is concluded that the stiffness difference ratio of the anchored coupling style is much larger than the direct coupling style. For the anchored diamond coupling TFG, the SDR is 16.08 times larger than the direct coupling one and is 5.46 times larger than the anchored ring coupling one.

### 4.2. Simulations

To analyze the vibration output, three architectures are designed. One is the completely symmetrical structure, and the stiffness imbalance of another two are 0.97% and 1.83%, respectively, which can be achieved by intentionally increasing the spring width of the left tine in the sense direction.

In this simulation, the simulation software, the element type and the mesh division are the same as in our previous study [13]. The total number of the mesh is 266,280, which is determined by the accuracy and efficiency of the calculation. In order to guarantee the efficiency and accuracy of the calculation, the frequency of the type A and type B designs is swept from 4000 Hz to 11,000 Hz and from 3000 Hz to 5000 Hz, respectively. The frequency step is 4 Hz, which is determined by the bandwidth. The material used in every model is single-crystal silicon and the corresponding Young’s modulus is 169 GPa. The thickness of structure is 60 µm and the external acceleration is 1 g (9.8 m/s^2^) and Q-factor of 100 which is just an assumption. This assumption is within a reasonable range from a hundred or so at atmospheric pressure to hundreds of thousands under vacuum. The model parameters and the modal frequencies are listed in Table 1 and Table 2, respectively.

However, a vibrational output would be produced by the linear vibration because the stiffness is imbalanced due to the practical process imperfections. Then, the harmonic responses of these two types are analyzed, in which ε is 0.97% and 1.83%.

**Figure 7:**
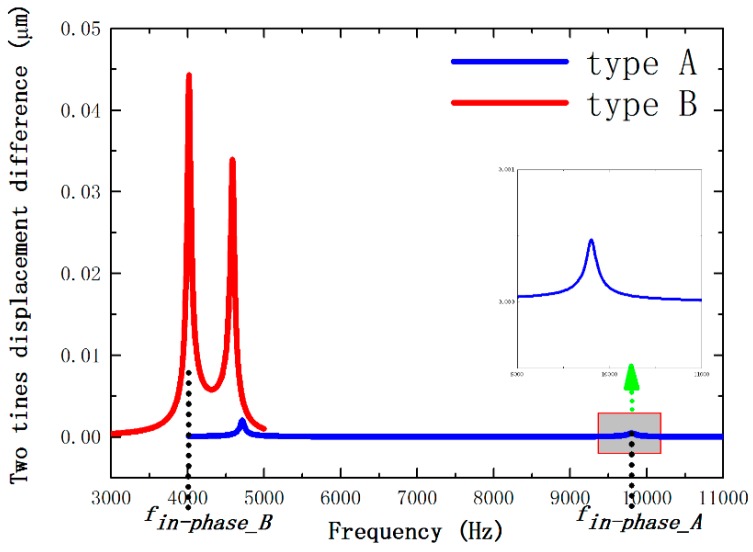
Displacement difference of 0.97% stiffness imbalance TFGs of two types.

As depicted in Figure 7, the two tines’ displacement difference can be acquired through the calculation of the simulation data. It can be observed that the modal frequency of two types is approximately equal in the anti-phase mode. For the displacement difference of Type A, it is much less than Type B and has a drastic reduction in the in-phase. Therefore, the Type A architecture is able to resist the linear vibration output much better than the Type B.

**Figure 8:**
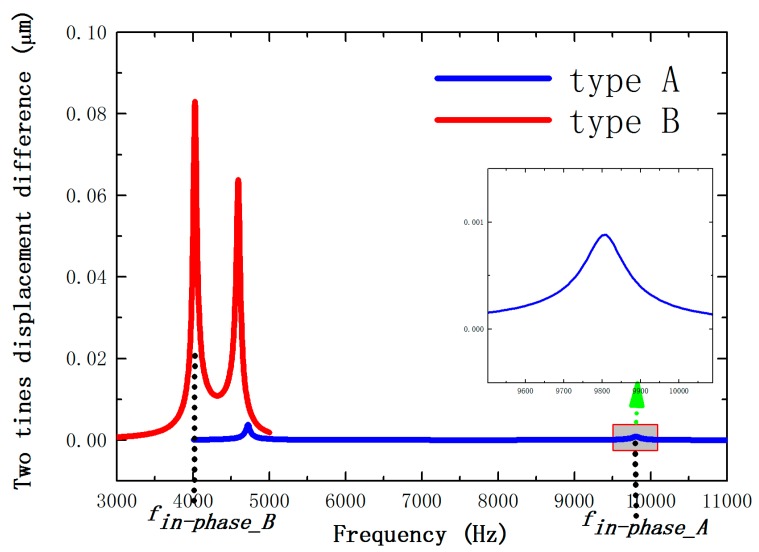
Displacement difference of 1.83% stiffness imbalance TFGs of two types.

The simulation with 1.83% stiffness imbalance is also carried out to analyze the impact of ε on the output. From Figure 8, it is seen that the vibration output becomes larger as ε increases.

### 4.3. Numerical and Theoretical Comparisons

The displacement difference between two masses is computed through the analytical expressions Equations (10) and (13). The theoretical and numerical values of Type A and Type B are then compared and the error rates are obtained, as indicated in Table 3.

From Table 3, one may figure out that theoretical results are consistent with simulations under small stiffness imbalances, which verifies the theoretical model we propose. To verify the theoretical modeling with a simplified spring, an analytical analysis is conducted on the previous study [16]. The comparisons with theoretical and numerical values are shown in Table 4. The type C denotes the anchored ring coupling structure while the type D denotes the direct ring coupling one. Therefore, the theoretical model can be very effective for anchored coupling structures. It is concluded that the displacement difference is inversely proportional to SDR and proportional to ε. Meanwhile, the theoretical values of displacement difference of type A and type B are also compared as depicted in Table 5, as well as type A and type C shown in Table 6.

**Figure 9:**
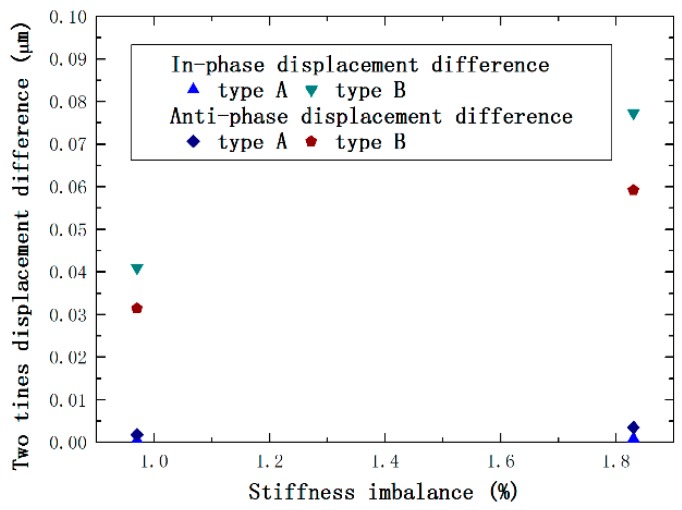
In- and anti-phase displacement difference of 0.97% and 1.83% stiffness imbalanced coupled tines of two types.

Table 5 shows that the displacement difference of type A is much less than for type B. Specifically, it is decreased by 94.1% and 99.0% compared to type B and is reduced by 82.6% and 93.2% compared to type C, in the anti- and in-phase modes, respectively. Additionally, the vibration output becomes larger as ε increases. Consequently, it is figured out the output of the anchored diamond coupling structure is much smaller than the direct coupling style due to the much larger stiffness difference ratio. To depict this conclusion better, the curve of the displacement difference of Type A and Type B and ε is drawn, as represented in Figure 9.

## 5. Discussion

From the theoretical analysis of the anchored coupling tuning fork gyroscopes, it is concluded that the anti-phase vibration output is proportional to ε and inversely proportional to η. To reduce ε, one way is to increase the beam widths and another one is to design a control circuit to suppress the stiffness imbalance. However, increasing the beam widths will reduce the scale factor and a stiffness-match circuit induces more complexity. Therefore, it is a more practical approach to increase the stiffness difference ratio only by changing the style of the coupling beam without sacrificing the sensitivity.

Actually, the deformation styles of the anchored coupling beam are different in the in- and anti-phase modes. In the anti-phase mode, the beam is bent under a lateral load while the beam is tensile (compressed) under an axial load in the in-phase described in Figure 5, which causes the stiffness in the in-phase mode is an order of magnitude larger than the anti-phase, so it can be believed that the vibration output of the anchored coupling style will be lower than the direct coupling style because of a larger stiffness difference ratio.

The analytical model we propose can be used for both anchored coupling TFGs and direct coupling TFGs. In our previous study [13], the analytical solutions of the vibration output of the direct coupling TFGs are calculated by using the matrix perturbation technique, which has been verified through our FEM simulations and experimental tests by other researchers [12]. The analytical expressions are in accordance with Equations (16) and (17), which proves the proposed analytical model in this paper. The analytical model is also valid to evaluate the vibration output of anchored coupling TFGs and only some of the parameters are different. The primary difference between the two types of TFGs is the coupling stiffness in the in-phase mode. The coupling stiffness in the in-phase is zero in the direct coupling one and the anchored coupling one is larger, which causes that the in-phase mode frequency is much higher than the anti-phase (improved mode ordering) and that the stiffness difference ratio is larger than the direct coupling one (reduced vibration sensitivity). In future studies, the anchored coupling TFGs we proposed will be fabricated and verified experimentally.

If someone proposes a cross structure, or anything similar, our modeling as presented in this paper will also be effective. Perhaps, some new structures will be designed to provide a higher stiffness difference ratio in the future. We believe that our theoretical model can be valid still.

## 6. Conclusions

In this paper, a new micromachined TFG with an anchored diamond coupling is proposed to investigate the corresponding mode ordering and the vibration sensitivity. The proposed TFG optimizes the sense-mode through use of an anchored diamond coupling beam, enabling the in-phase mode frequency to be 108.3% larger than the anti-phase one. A new theoretical model is established to investigate the vibration sensitivity of micromachied TFGs with anchored coupling. The coordinate transformation method is used to compute the dynamic response caused by the common-mode vibration, which coincides with the FEM simulations. The analytical solutions show that the anti-phase vibration output is inversely proportional to the stiffness difference ratio and proportional to the stiffness imbalance. Additionally, the stiffness difference ratio of the two types of TFGs is obtained by the FEM simulations. The simulations demonstrate that the stiffness difference ratio of the anchored diamond coupling structure is much larger than the direct coupling one and the SDR of the anchored diamond coupling structure is 16.08 times larger than the direct coupling one while the vibration output is reduced by 94.1% in the anti-phase sense mode frequency. In comparison with the anchored ring coupling structure, the SDR of the anchored diamond coupling one is 5.46 times larger than the anchored ring coupling one, while the vibration output is reduced by 82.6%. Consequently, the anchored diamond coupling TFG is able to structurally offer a higher stiffness difference ratio to improve the mode ordering and tremendously reject the vibration output without sacrificing any sensitivity.

## Figures and Tables

**Table 1:** Model parameters used in the simulation models.

Parameters	Value	Parameters	Value
Sense-mode mass	1.3738 × 10^−6^ Kg	Structural thickness	60 µm
Structure type	Type A/Type B	Sense-mode Q	100
Stiffness imbalance	0.97%/1.83%	Common acceleration	9.8 m/s^2^
Springs stiffness *k*	748.5 N/m	Stiffness difference ratio η_a_/η_b_	11.80/0.73

**Table 2:** In-phase and anti-phase modal frequencies of all designed models.

ε (%)	In-Phase Modal Frequency (Hz)		Anti-Phase Modal Frequency (Hz)	
	Type A	Type B	Type A	Type B
0	9799.6	4007.1	4705.3	4578.1
0.97	9803.0	4015.7	4712.7	4585.9
1.83	9806.0	4023.2	4719.3	4592.9

**Table 3:** Comparisons with theoretical and numerical values of Type A and Type B.

	Type	Type A			Type B		
ε		Theoretical Value	Simulation Value	Error Rate	Theoretical Value	Simulation Value	Error Rate
In-phase displacement difference (µm)	0.97%	0.000425	0.000456	7.33%	0.0410	0.0444	8.20%
	1.83%	0.000802	0.000853	6.40%	0.0773	0.0832	7.62%
Anti-phase displacement difference (µm)	0.97%	0.00184	0.00199	7.89%	0.0314	0.0340	8.17%
	1.83%	0.00347	0.00372	7.08%	0.0592	0.0636	7.35%

**Table 4:** Comparisons with theoretical and numerical values of Type C and Type D.

	Type	Type C			Type D		
ε		Theoretical Value	Simulation Value	Error Rate	Theoretical Value	Simulation Value	Error Rate
In-phase displacement difference (µm)	0.97%	0.00627	0.0068	6.14%	0.0525	0.0561	6.42%
	1.83%	0.0118	0.0125	5.60%	0.0986	0.105	6.10%
Anti-phase displacement difference (µm)	0.97%	0.0106	0.0114	7.02%	0.0423	0.0453	6.62%
	1.83%	0.0200	0.0212	5.66%	0.0795	0.0844	5.81%

**Table 5:** Comparisons of displacement difference in analytical values of Type A and Type B.

ε (%)		Type A	Type B	Reduced Rate
In-phase displacement difference (µm)	0.97	0.000425	0.0410	99.0%
	1.83	0.000802	0.0773	
Anti-phase displacement difference (µm)	0.97	0.00184	0.0314	94.1%
	1.83	0.00347	0.0592	

**Table 6:** Comparisons of displacement difference in analytical values of Type A and Type C.

ε (%)		Type A	Type C	Reduced Rate
In-phase displacement difference (µm)	0.97	0.000425	0.00627	93.2%
	1.83	0.000802	0.0118	
Anti-phase displacement difference (µm)	0.97	0.00184	0.0106	82.6%
	1.83	0.00347	0.0200	
